# Supplementary material for: Acquired tick resistance in Peromyscus leucopus alters Ixodes scapularis infection
Source: Infect Immun. 2025 Sep 3;93(10):e00246-25. doi: 10.1128/iai.00246-25 (PMC12519807; doi:10.1128/iai.00246-25)
Supplement: Supplemental figures — Figures S1 to S5. [file iai.00246-25-s0001.docx]

**
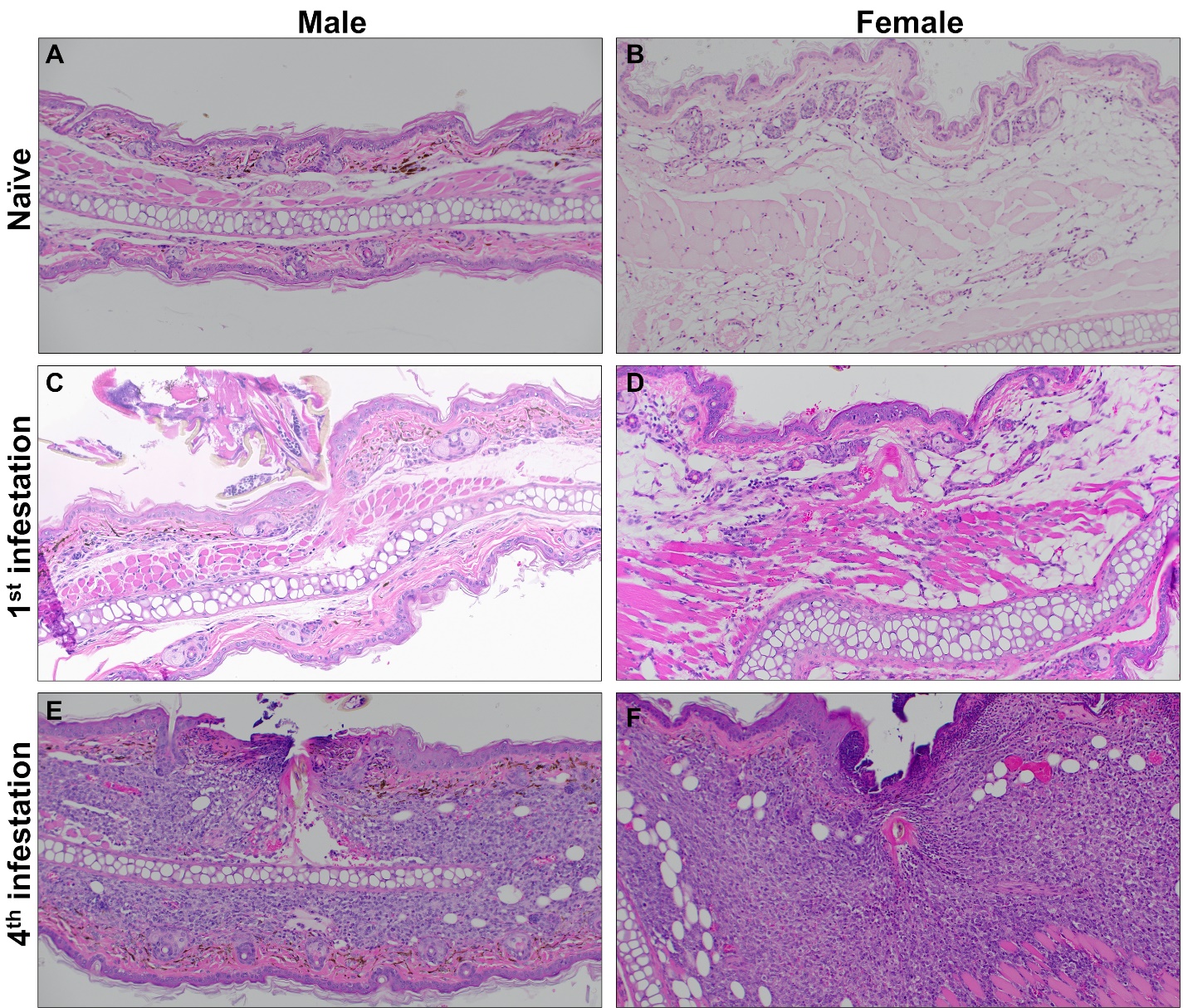
**

**Supplemental Figure 1. Increasing inflammation at larval attachment sites with previous tick exposure, original raw images.** Original histology images of male and female mouse biopsies from (A-B) naïve pinna, and (C-F) larval attachment sites from (C-D) a primary infestation and (E-F) a quaternary infestation. H&E stain.

**
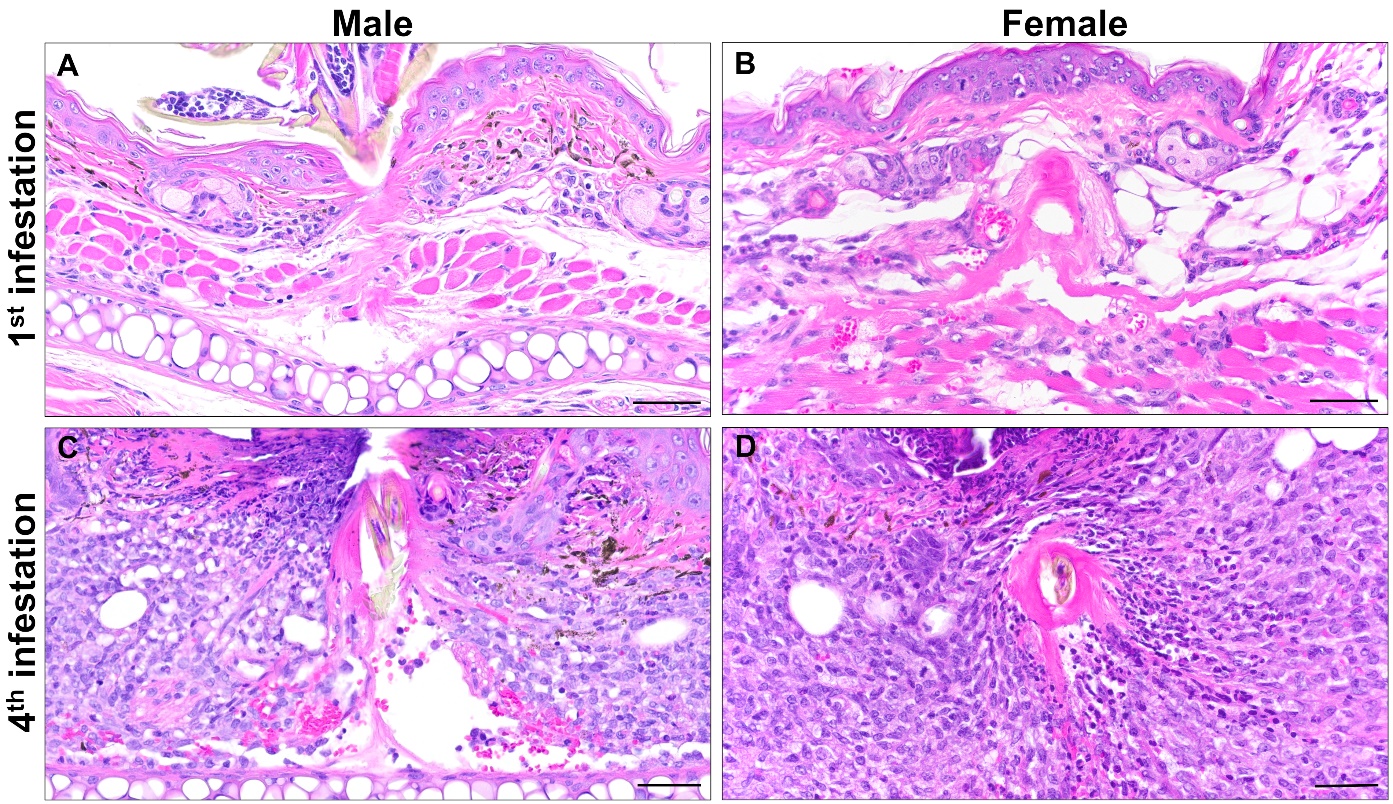
**

**Supplemental Figure 2. High magnification images of larval attachment sites on tick-naïve and tick-sensitized mice**. Histological examination of male and female mouse biopsies from (A-B) primary infestations, and (C-D) quaternary infestations. H&E stain. Bar = 50 μm.


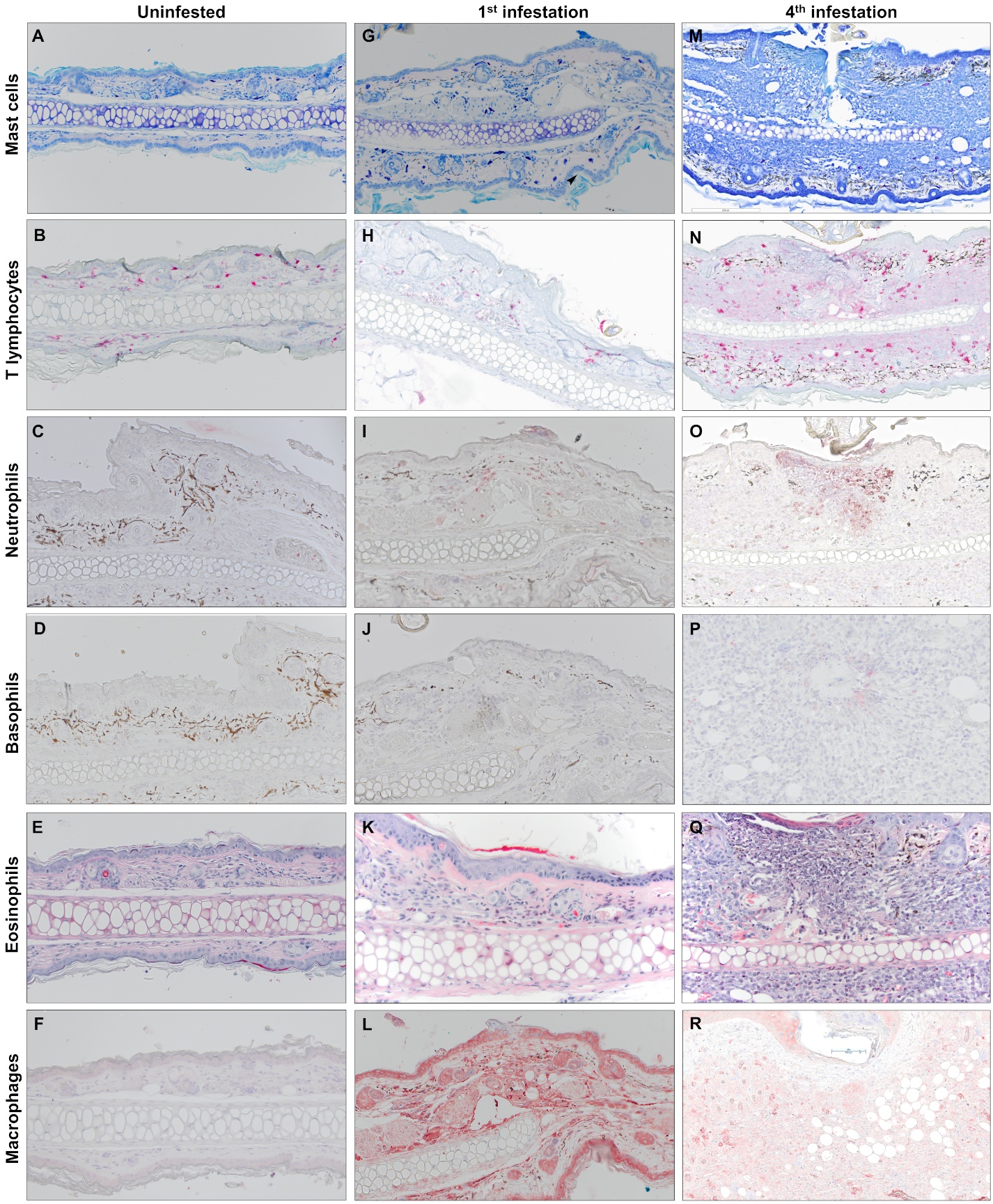


**Supplemental Figure 3. Serial infestation of male *P. leucopus* with larval *I. scapularis* elicits eosinophil, neutrophil, basophil, T lymphocyte, and macrophage infiltrates, original raw images.** Original images of special stains and immunohistochemistry performed on naïve skin and at primary and quaternary larval attachment sites in male mice. (A,G,M) Mast cells are visualized with toluidine blue stain. (B,H,N) T lymphocytes were visualized by immunohistochemistry against CD3 (cluster of differentiation 3). (C,I,O) Neutrophils are visualized by immunohistochemistry against Myeloperoxidase. (D,J,P) Basophils were visualized by immunohistochemistry against Mcpt8 (mast cell protease 8). (E,K,Q) Eosinophils are visualized by Luna stain. (F,J,R) Macrophages were visualized by immunohistochemistry against ionized calcium-binding adaptor molecule 1 (IBA1).


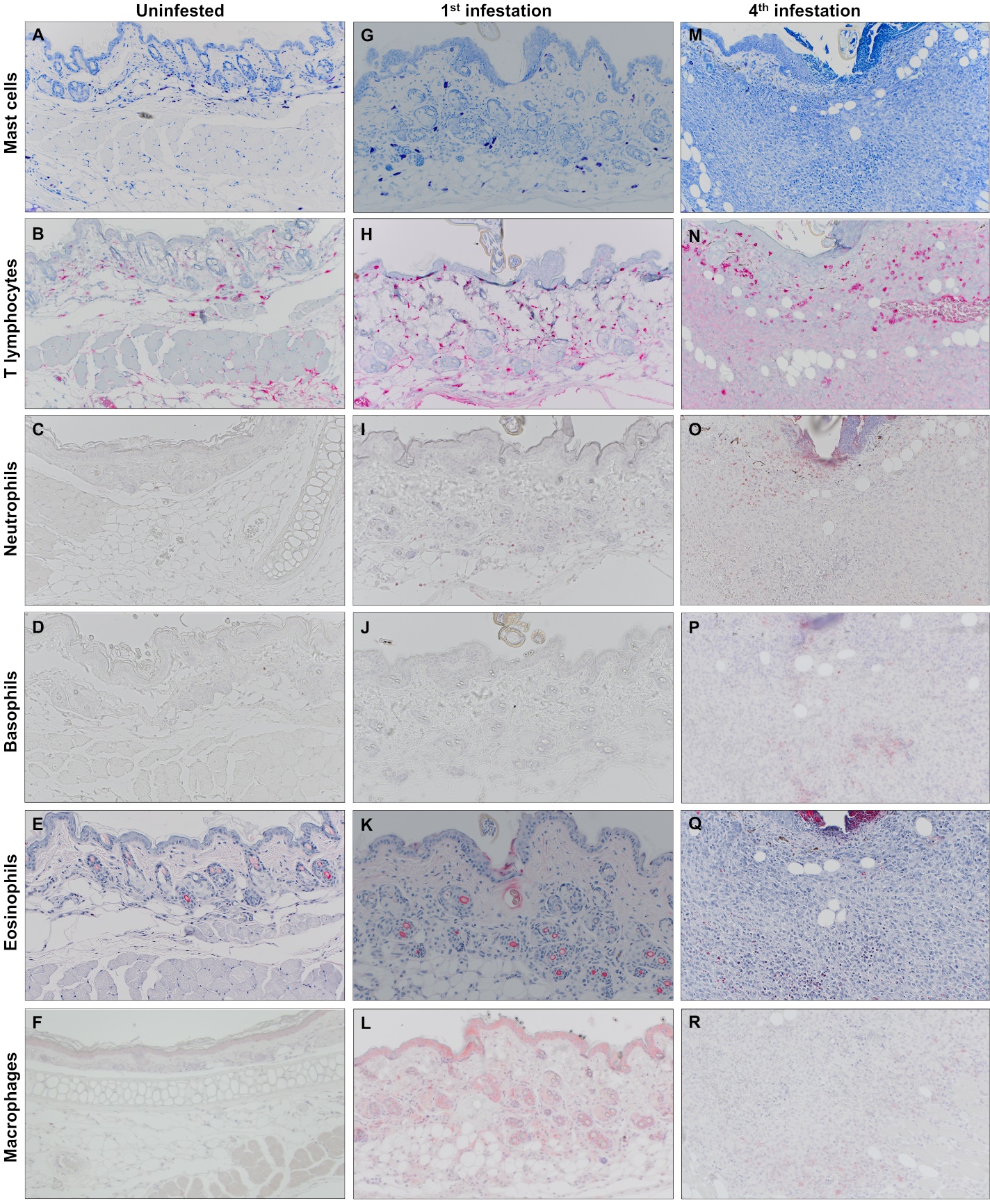


**Supplemental Figure 4. Serial infestation of female *P. leucopus* with larval *I. scapularis* elicits eosinophil, neutrophil, basophil, T lymphocyte, and macrophage infiltrates, original raw images.** Original raw images of special stains and immunohistochemistry performed on naïve skin and at primary and quaternary larval attachment sites in female mice. (A,G,M) Mast cells are visualized with toluidine blue stain. (B,H,N) T lymphocytes were visualized by immunohistochemistry against CD3 (cluster of differentiation 3). (C,I,O) Neutrophils are visualized by immunohistochemistry against Myeloperoxidase. (D,J,P) Basophils were visualized by immunohistochemistry against Mcpt8 (mast cell protease 8). (E,K,Q) Eosinophils are visualized by Luna stain. (F,J,R) Macrophages were visualized by immunohistochemistry against ionized calcium-binding adaptor molecule 1 (IBA1).


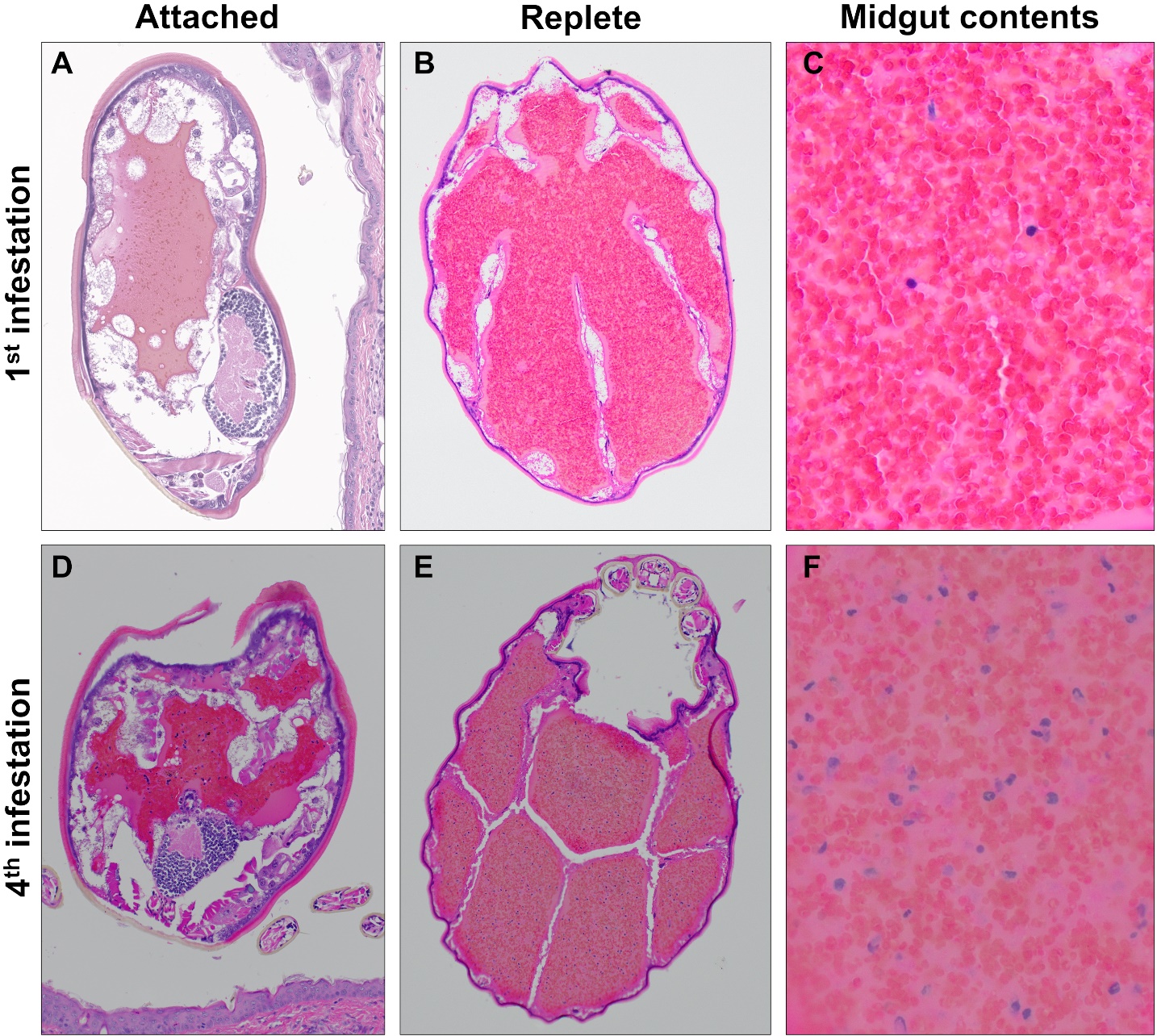


**Supplemental Figure 5. Larvae fed on tick-sensitized hosts ingest more host leukocytes, original raw images.** Original raw histologic images of attached and replete larvae. (A-C) Histology of larval cross sections from ticks fed on a naïve mouse (1^st^ infestation) at 3-days post-attachment (A) and after repletion (B). (C) Nucleated cells shown in the midgut at high magnification. (D-F) Histology of larval cross sections from ticks fed on a sensitized mouse (4^th^ infestation) at 3-days post-attachment (D) and after repletion (E). (F) Nucleated cells with multilobulated or fragmented nuclei are shown in the midgut at high magnification. H&E stain.
